# The Effect of Screen Habits and Alternative Activities on Tactile Exploration Skills in 6- to 36-Month-Old Toddlers

**DOI:** 10.3390/children11081027

**Published:** 2024-08-22

**Authors:** Estelle Gillioz, Edouard Gentaz, Fleur Lejeune

**Affiliations:** 1 Department of Psychology, University of Geneva,1205 Geneva, Switzerland; edouard.gentaz@unige.ch (E.G.); fleur.lejeune@unige.ch (F.L.); 2 Swiss Center for Affective Sciences, University of Geneva, 1205 Geneva, Switzerland; 3 Centre National de la Recherche Scientifique (CNRS), 75116 Paris, France

**Keywords:** toddlers, development, screen habits, tactile exploration skills, visuo-tactile exploration

## Abstract

Background/Objectives: With the rising ubiquity of digital media and screens in everyday life, toddlers are increasingly exposed to different screens from an early age (i.e., television, computer, tablet, phone). However, few studies have examined the effect of these screens on toddlers’ perceptual development. Since tactile exploration skills are necessary for environmental discovery and overall development, the current research investigates the links between screen-use habits and the tactile exploration skills (with visual control) of 6- to 36-month-old toddlers. Methods: The study involved observing the interactions of 135 toddlers with various objects and assessing the complexity of their visuo-tactile exploration strategies through two original experimental tasks. Data concerning screen habits and other relevant factors, such as socio-economic level, were collected using a parental questionnaire. Results: Toddlers with greater screen exposure time demonstrated weaker tactile exploration skills and employed less age-appropriate exploration strategies. Socio-economic factors and parental engagement in alternative activities significantly influenced these developmental outcomes. Conclusions: These findings emphasize the importance of reducing screen time and promoting interactive co-viewing and alternative activities to mitigate the negative effects of screen exposure. Further longitudinal research is needed to determine the long-term impacts of early screen exposure on tactile exploration and overall psychological development.

## 1. Introduction

Digital media and screen use have become increasingly ubiquitous thanks to technological advances and accessibility, and they are now an integral part of our daily lives [1]. They are used in an ever-increasing variety of contexts and have even become necessary for everyday tasks such as paying bills, working, and keeping in touch with loved ones and family. Families in many countries are now multi-equipped, with an average of seven screens per household (for examples, cf. Ref. [2] in France; cf. Ref. [3] in Switzerland). In this context, children are provided with more opportunities to be exposed to digital devices from an early age. The age of initial screen exposure is becoming increasingly younger [4,5], while exposure times are tending to increase [6,7]. Toddlers between 0 and 3 years are exposed on average six days a week for between thirty minutes and three hours a day, and these numbers increased following the COVID-19 pandemic [8,9,10]. Children are most often left alone in front of screens: parents or caregivers generally use media and other devices as a babysitter or as a calming tool for children [11]. In addition, a large majority of parents in Switzerland say they spend time in front of screens while their infant is present in the room [3]. Toddlers are therefore subjected to background screen exposure, while parents and/or siblings watch their shows on television or use screens in front of them. However young children need to interact with their caregivers, create a secure attachment, and be able to explore their environment in order to develop harmoniously [12]. They need to be able to look, listen, touch, smell, and use their five senses to open up to the world around them and their immediate environment, and this happens mainly through tactile exploration [13,14].

Touch is the first sense to develop: fetuses already adopt behaviors that can be considered as the early precursors of reaching-to-grasp and perform sensory-motor coordination tasks in utero, such as sucking their thumbs or touching their faces and hands [15,16]. After birth, infants first explore their own bodies, then their exploration strategies develop and multiply with age, gradually focusing on the objects around them [17,18]. At first, they spend most of their time putting objects into their mouths, shaking them, and throwing them. As they become able to coordinate eye, hand, and body movements, they engage in more technical and complex exploration behaviors [19,20]. At this point, around six month of age, the infant’s visual fixation and tactile discovery activities become synchronous [21,22]. The wariness effect described by Schaffer et al. [23] supports these findings: at nine months old, toddlers take longer to manually grasp and explore a new object than a familiar ones, demonstrating a link between perceptual information and motor behaviors. Moreover, eye–hand coordination is linked to the development of fine motor skills [20], a prerequisite for motor actions. These motor actions enable children to develop general and more specific knowledge about objects. This knowledge can provide them with important information to make their actions more precise and effective [24,25]. They will begin to turn objects to explore their contours visually, to caress them to feel their textures, to press them to discover their volume, or to transfer them from one hand to the other to test their physical properties [26]. These skills evolve enormously over the first years of life [27] and are necessary for children’s overall psychological development. There is a large consensus regarding the benefits of object interactions for infant development. Object interactions facilitate increasingly complex, flexible, and controlled motor actions [28] and enhance children’s physical capacities, as well as symbolic capacities, when objects are used to replace other things in pretend play [29,30]. All these processes enable children to engage in a variety of everyday activities essential for integrating into society, developing psychosocial skills, and achieving personal fulfillment [31]. Moreover, visuo-manual interactions with objects support perceptual development, spatial understanding, problem-solving, and memory, as well as promote learning about the physical properties of objects (including the understanding that objects are three-dimensional [32,33];). All these skills are necessary for success at school: children who spend more time exploring as toddlers are in fact those who show better academic performance once they reach adolescence [34]. On the contrary, children who show delays in tactile exploration or use less-developed exploration strategies are at greater risk of developing cognitive, motor, and language delays, as they miss opportunities to explore and learn from their environment [35]. Visuo-tactile exploration is therefore essential to children’s overall development, as it contributes to cognitive, sensory, identity, social, and emotional development [36]. Supporting its development is consequently necessary, especially as this is a very early skill in a toddler’s development, and it evolves rapidly over the first three years of life [37,38,39].

While sitting in front of screens, children’s learning opportunities and interactions are reduced. Empirical evidence suggests that, in most cases, screen exposure is associated with poorer mental and physical health, as well as with delayed cognitive, emotional, language, and academic development [4,40,41,42,43,44,45,46]. These studies mainly analyze solitary, passive screen use (e.g., watching television or a video on a computer). It is nevertheless necessary to study this exposure in more detail, examining the various confounding factors and the different screens used by toddlers. For example, Hu et al. [41] found a negative link between passive screen use and children’s cognitive development, whereas no such link was found when children were actively using interactive screens (i.e., media that require interactive input, e.g., tablets, phones, computers). It is even possible to find positive links based on the content being viewed. Madigan et al. [43], for example, found a positive correlation between the viewing of educational programs and language development in young children. In addition, many of these negative effects have been shown to be moderated by factors such as parenting style [47] or the practice of interactive co-viewing during exposure [48,49,50], and may disappear when confounding factors, including socio-economic level, are considered [51]. In any case, it seems that the time spent by toddlers in front of screens is time lost to other activities. This is defined in the literature as the displacement hypothesis: it postulates that extended screen use would reduce the time and opportunities available for the interpersonal experiences necessary for children’s socio-emotional and cognitive development, as well as the time they could devote to exploring their environment. Alone in front of a screen, toddlers do not use their body and hands to discover and interact with surrounding objects, an activity which enables them to improve their complex, flexible, and controlled motor actions. This is the case, even when using an interactive screen (e.g., tablets): compared with actions that involve grasping objects, the involvement of muscle strength, coordination, and dexterity are relatively lower when interacting with screens [52]. Moreover, screens are notorious for being real distracters: the colors, movements, and sounds they broadcast capture the toddler’s attention and disrupt the activity in progress [53,54]. When a screen is switched on in their presence, they are consequently less attentive to their own bodies and to the objects around them. Taken together, all these factors could have an impact on the development of tactile exploration skills in young children. Nevertheless, empirical evidence relating early screen exposure in toddlerhood to delays in cognitive, socio-emotional, and sensory development are currently lacking.

It therefore seems appropriate to consider the effects of screens on the development of 6- to 36-month-old toddlers’ visuo-tactile exploration. The reasons for this study are as follows: Firstly, children in front of a screen are not exploring their environment, and the amount of time they spend there is constantly increasing. Secondly, background exposure also interferes with children’s exploration, cutting them off from their discovery activity or the game they are currently playing [54]. Finally, there is as yet no empirical evidence linking the early exposure of toddlers to screens with the development of their visuo-tactile exploration skills, even though this is an ability essential to children’s overall psychological development, which develops rapidly and early in life.

This study aims to determine the links between screen-use habits and the development of 6- to 36-month-old toddlers’ visuo-tactile exploration skills, as well as to investigate possible links with different factors such as the practice of interactive co-viewing during exposure, alternative activities carried out at home, and the families’ socio-economic level. One or two original experimental tactile tasks (with visual control) were proposed as a function of age. For this purpose, we hypothesized that: (1) Toddlers’ visuo-tactile exploration skills and the efficiency of the exploration strategies used increase with age; (2) Toddlers with greater screen exposure time have weaker visuo-tactile exploration skills and use more strategies with are unsuited to their age; (3) The family environment in which toddlers grow up plays an important role in their development, e.g., factors such as the practice of interactive co-viewing during exposure, the alternative activities carried out at home, and the socio-economic level of the household influence the development of the child’s visuo-tactile exploration skills.

## 2. Materials and Methods

### 2.1. Participants

In this study, we observed 135 infants, divided into three groups according to the tasks proposed and the recommendations for screen use: 40 between 6 and 18 months old (*M* = 11.5 months, *SD* = 3), 34 between 19 and 24 months old (*M* = 21 months, *SD* = 2.5), and 61 between 25 and 36 months old (*M* = 31 months, *SD* = 4). This separation allows for a more detailed analysis of the development of tactile exploration skills and enables inter-group comparisons. Families were recruited through a child daycare facility in French-speaking Switzerland, with no exclusion criteria. However, parents were asked to report in the questionnaire whether their toddler had any developmental deficits or delays, which was not the case for the toddlers included in our sample. The average families’ socio-economic level is confirmed as entrance into the Swiss upper-middle class, according to a socio-economic position index calculated from the age, the level of education, and the professional category of both parents (*M* = 69.85, *SD* = 12.965; [55]), and all parents were native French speakers.

### 2.2. Procedure

This study was part of a larger project investigating links between screen habits and several aspects of child development, approved by the Ethics Committee of the Faculty of Psychology and Educational Sciences at the University of Geneva. The principal investigator explained the complete procedures to all parents, from whom written informed consent was obtained before the children were enrolled in the study. The parents also completed the online questionnaire before we met the toddlers. All assessments were filmed and carried out individually with each toddler and his parent or his accompanying daycare staff member. During the assessment, the toddlers were asked to perform various tasks, including two experimental tasks assessing tactile exploration. The total duration of the encounter was approximately thirty minutes.

### 2.3. Materials and Measures

#### 2.3.1. Questionnaire Regarding Screen Habits and Other Daily Activities

Toddlers’ screen habits and other daily activities were measured via different questions in a questionnaire to be completed online by parents (Qualtrics, Provo, UT, USA) before meeting the toddlers to carry out all the experimental tasks in the laboratory. The questionnaire was specifically designed for this study, enabling us to quickly and non-invasively gather information on the use of screens in households, particularly the use of screens by toddlers. It allows us to obtain different measures, including: (1) Direct screen exposure time (in minutes per day for a full week)—parents were required to report how many minutes a day their toddler spends in front of screens during a typical weekday and over the weekend; (2) Background exposure time (in minutes per day)—parents were required to report how many minutes per day they or someone else use screens in front of the child; (3) Total screen exposure time (in minutes per day)—this was obtained by the addition of the direct screen exposure time and the background exposure time; (4) Interactive co-viewing—parents were required to indicate whether or not they engage in interactive co-viewing with their infant during exposure; (5) Daily activities carried out at home—parents were required to report all the others activities their toddler engaged in at home. The answers provided were separated into six categories according to the games and activities performed—reading, sensory awareness games, manual construction games, imitation games, outdoor games, and cultural activities. This makes it possible to calculate the total number of different alternative activities carried out during the day.

#### 2.3.2. Tactile Exploration Tasks

To assess toddlers’ tactile exploration skills, two original, never-before-used experimental tasks were proposed in laboratory: the “three objects task” and the “cube task”. Both involved object discovery and visuo-manual control, but the former measured the toddlers’ tactile exploration skills more precisely in terms of the visuo-tactile exploration strategies used to discover objects, while the latter focused more on the relevance of visuo-tactile exploration in terms of the part of the object being explored.

##### Three Objects Task in 6–18-Month- and 19–24-Month-Old Toddlers

**Figure 1:**
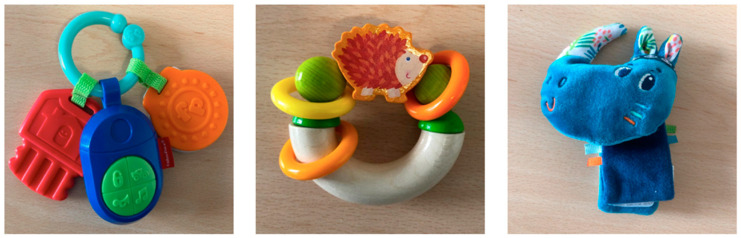
The plastic keys, the wooden rattle, and the wrist cuddly toy (Fisher Price brand^©^, East Aurora, NY, USA).

The three objects task consisted of the exploration of three distinct objects: a wooden rattle, a wrist cuddly toy, and plastic keys (Figure 1). These three objects were different in terms of appearance, textures, and tactile exploration strategies they could induce, which is why they were employed in this study. Since these objects were designed for the very young, and a preliminary study showed that 25- to 36-month-old toddlers were not interested at all; only children between 6 and 18 months and between 19 and 24 months old performed this task. No specific oral instructions were given to toddlers. They were seated at a table on the lap of their caregiver or their daycare staff member, and the different objects were placed on the table in front of them by the experimenter, who did not say anything. The objects were within easy reach of the toddlers, who could observe and explore the objects as they wished, using their two hands. Since a preliminary study showed that toddlers’ interest in these different objects diminished after a maximum of three minutes, only the first three minutes of exploration were analyzed in this study. The measures were the total exploration time in seconds, the number of different strategies used (including putting it in the mouth, caressing, throwing, pressing, turning and observing, and shaking), the total number of strategies used, as well as the number of times each of the six exploration strategies was used during the first three minutes of exploration.

##### The Cube Task in 6–18-Month, 19–24-Month, and 25–36-Month-Old Toddlers

**Figure 2:**
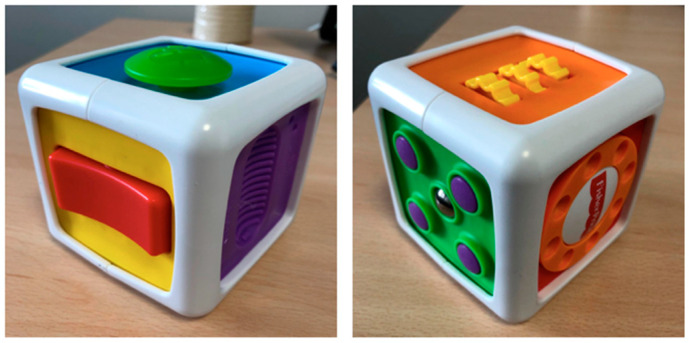
The multifaceted plastic cube (Fisher Price brand^©^).

The cube task comprised the exploration of one multifaceted plastic cube, chosen because it is a sufficiently familiar object for children and is suitable for all ages. Each side of the cube features a specific activity and requires varying degrees of tactile exploration skills: for example, the orange side displayed three inlaid yellow cogs that turned on themselves, producing a mechanical noise if the toddlers managed to turn them, while the purple face had a special linear texture that was revealed via touch, requiring no special skills (Figure 2). Each of the three age groups performed this task. No specific oral instructions were given to toddlers. They were seated at a table on the lap of their caregiver or their daycare staff member, and the multifaceted plastic cube was placed on the table in front of them by the experimenter, who did not say anything. They could observe and explore the cube as they wished, using their two hands. For similar reasons as those applicable to the three objects task, only the first two minutes of exploration were analyzed for this task. The measures were the total exploration time in seconds, the number of different faces explored; the total number of faces explored; and the exploration time, in seconds, for each of the six faces separately (orange, purple, green, yellow, red, and blue) during the first two minutes of exploration.

### 2.4. Data Analysis

The analyses were performed after all the data were collected. To ensure inter-observer reliability, two psychologists previously trained in the rating of these tasks scored every variable separately. In the event of disagreement, the videos were discussed and recoded by both coders together.

To identify the various independent variables concerning screen habits, toddlers in each age group were divided into two groups. Concerning the direct screen exposure time, we firstly aligned ourselves with the accepted international guidelines (i.e., no screens before the age of 18 months, and less than one hour per day thereafter [56]), as is the case in several studies (e.g., Refs. [57,58]). Toddlers between 6 and 18 months old were therefore separated into two groups according to whether they had already been exposed to screens or not: those who were not yet directly exposed to screen were assigned to group 0 (GR0), and those who had already been exposed were assigned to group 1 (GR1). However, since most 19- to 24-month-old toddlers and 25- to 36-month old toddlers adhered to the international guidelines, we felt it more appropriate to base our analysis on actual screen time and thus, divided these two groups according to the average total minutes per day they spent in front of screens. Toddlers who were exposed to more screen time than the average were assigned to group 1 (GR1), and toddlers who were exposed to less than or equal to the average screen time were assigned to group 0 (GR0). Concerning the background screen exposure time and the total screen exposure time, no guidelines were specified. We also decided to divided toddlers in each age group into two groups, according the average of total minutes they spent in front of screens per day. Toddlers who were exposed to more than the average were assigned to group 1 (GR1), and toddlers who were exposed less than or equal to the average were assigned to group 0 (GR0). Parents also indicated whether or not they engaged in interactive co-viewing with their infant during exposure. Toddlers who experienced interactive co-viewing were assigned to group 1 (GR1), and those who did not were assigned to group 0 (GR0), except for toddlers between 6 and 18 months old, who never engaged in interactive co-viewing.

We conducted the data analysis using IBM SPSS Statistics, version 29.0.2.0. As our sample sizes are relatively small per subgroup, the central limit theorem does not apply, and we performed Kolmogorov–Smirnov tests to check the normality of our data before proceeding with our main statistical analyses. We therefore ran independent samples *t*-tests whenever possible (i.e., when the data on the variable of interest are normally distributed in each subgroup), or Mann–Whitney tests when the data failed to meet the assumptions of parametric analysis, using screen habits (direct screen exposure time, background exposure time, total screen exposure time, and interactive co-viewing) as independent variables and the different tactile exploration scores as dependent variables. Concerning the total number of alternative activities carried out daily at home and our dependent variables of interest, we conducted Pearson correlation tests. Finally, to control for the effect of socio-economic level, we first ran independent samples *t*-tests using, screen habits as independent variables and the socio-economic level as dependent variables. When a significant difference appeared between the groups, we performed ANCOVA evaluations using screen habits as fixed factors, the different tactile exploration scores as dependent variables, and the families’ socio-economic level as a covariate. A significance level of 0.05 was used for all statistical tests. We also set a trend significance level of 0.08.

## 3. Results

### 3.1. The Screen Habits as Function of the Degree of Exposure and the Age Groups

In Table 1, the direct screen exposure time, the background exposure time, and the total screen exposure time per day are presented for each subgroup.

### 3.2. Effect of Screen Habits and Alternative Activities on Tactile Exploration in 6- to 18-Month-Old Toddlers

Table 2 showed the scores obtained for the two tactile exploration tasks by each subgroup of toddlers between 6 and 18 months old, based on screen habits.

Three objects task. The results indicated that there were no significant differences between the total exploration time, the number of different strategies used, and the total number of strategies used, depending on the direct screen exposure time, the background exposure time, and the total screen exposure time in 6- to 18-month-old toddlers (all *ps* > 0.05). Whatever their screens habits, 6- to 18-month-old toddlers explored all three objects in the same way and used the same strategies. Similarly, the results showed no significant correlation between alternative activities carried out at home and the toddlers’ tactile exploration skills (all *ps* > 0.05).

Cube task. The results showed no significant differences between the total exploration time, the number of different faces explored, and the total number of explored faces, depending on the direct screen exposure time, the background exposure time, and the total screen exposure time in 6- to 18-month-old toddlers (all *ps* > 0.05). Similarly, the results showed no significant correlation between alternative activities carried out at home and the toddlers’ tactile exploration skills (all *ps* > 0.05).

By analyzing the exploration time for each face of the cube separately, the results indicated that toddlers who had not yet been directly exposed to screens explored the red face significantly longer (GR0: *M* = 5.07, *SD* = 7.4) than those already exposed to screens (GR1: *M* = 1.31, *SD* = 4.7); *U* = 103, *p* = 0.036.

### 3.3. Effect of Screen Habits and Alternative Activities on Tactile Exploration in 19- to 24-Month-Old Toddlers

Table 3 showed the scores obtained for the two tactile exploration tasks by each subgroup of toddlers between 19 and 24 months old, based on screen habits.

Three objects task. The results showed no significant differences between the total exploration time, the number of different strategies used, and the total number of strategies used, depending on the direct screen exposure time, the background exposure time, and the total screen exposure time, as well as with the practice or not of co-viewing (all *ps* > 0.05).

By analyzing the number of times each strategy was used separately, the results indicated that toddlers with a greater background exposure time used the shaking strategy more often (GR1: *M* = 1.82, *SD* = 2) than those with a shorter exposure time (GR0: *M* = 0.48, *SD* = 0.9); *U* = 189.5, *p* = 0.019. Moreover, they also tended to use the throwing strategy more often (GR1: *M* = 2, *SD* = 3.1) than those with a shorter background exposure time (GR0: *M* = 0.22, *SD* = 1); *U* = 178, *p* = 0.06. These results were similar for the total exposure time: toddlers with a greater total exposure time used the shaking strategy more often (GR1: *M* = 1.67, *SD* = 2) than those with a shorter exposure time (GR0: *M* = 0.5, *SD* = 1)—*U* = 187.5, *p* = 0.044—and they also tended to use the throwing strategy more often (GR1: *M* = 1.83, *SD* = 3; GR0: *M* = 0.23, *SD* = 1.1); *U* = 180.5, *p* = 0.08. The results also highlighted a correlation between the number of alternative activities carried out daily by toddlers and the use of the pressing strategy—*r* = 0.398, *p* = 0.032—as well as correlations that tended towards significance between the number of alternative activities and the use of the turn and observe strategy—*r* = 0.350, *p* = 0.063—and with the total number of strategies used—*r* = 0.346, *p* = 0.066. Toddlers who participated in a greater number of different alternative activities during the day used the pressing strategy more often and seemed to use the turn and observe strategy more often, as well as to employ more strategies in total.

Cube task. The results showed no significant differences between the total exploration time, the number of different faces explored, and the total number of explored faces, depending on the direct screen exposure time, the background exposure time, and the total screen exposure time, as well as with the practice or not of co-viewing (all *ps* > 0.05). Similarly, the results showed no significant correlation between alternative activities carried out at home and the toddlers’ tactile exploration skills (all *ps* > 0.05).

There was only a difference that approached statistical significance in regards to the total exploration time depending on direct screen exposure time: toddlers with a greater direct screen exposure time seemed to exhibit a greater total exploration time (GR1: *M* = 61.25, *SD* = 17.6) than those with a shorter direct screen exposure time (GR0: *M* = 43.35, *SD* = 28.7), *t*(32) = −1.661, *p* = 0.053.

### 3.4. Effect of Screen Habits and Alternative Activities on Tactile Exploration in 25- to 36-Month-Old Toddlers

Table 4 shows the scores obtained for the two tactile exploration tasks by each subgroup of toddlers between 25 and 36 months old, based on screen habits.

Cube task. The results showed significant differences between the total number of explored faces, depending on the background exposure time and the total screen exposure time: toddlers with greater background (GR1: *M* = 6.74, *SD* = 1.8) and total exposure times (GR1: *M* = 6.62, *SD* = 1.9) explored more faces in total than those with shorter background (GR0: *M* = 5.05, *SD* = 3.2) and total exposure times (GR0: *M* = 5.03, *SD* = 3.2); respectively, *U* = 555, *p* = 0.015, and *U* = 573.5, *p* = 0.019. However, these groups differed in their socio-economic level: the results indicated significant differences between the socio-economic level of the toddlers’ families and the background exposure time—(GR0: *M* = 73.98, *SD* = 8.5, GR1: *M* = 59.66, *SD* = 17.9), *t*(59) = 3.307, *p* = 0.003—and the total screen exposure time—(GR0: *M* = 74.55, *SD* = 7.8, GR1: *M* = 59.93, *SD* = 17.5), *t*(59) = 3.644, *p* = 0.001—and these latter results are no longer significant after controlling for this factor; respectively, *p* = 0.133 for background exposure time, and *p* = 0.162 for total screen exposure time.

By analyzing the exploration time for each face of the cube separately, the results indicated that toddlers with a greater direct screen exposure time explored the purple face significantly longer (GR1: *M* = 4.029, *SD* = 8.6) than those with a shorter exposure time—(GR0: *M* = 0.523, *SD* = 1.7), *U* = 471, *p* = 0.02—and this result also tended towards significance for the background exposure time (GR0: *M* = 1.27, *SD* = 5.3; GR1: *M* = 2, *SD* = 3.8; *U* = 476, *p* = 0.076). Moreover, the results indicated that toddlers who did not engaged in interactive co-viewing during screen exposure explored the orange face longer (GR0: *M* = 21.19, *SD* = 21.3) than toddlers who engaged in interactive co-viewing during exposure time—(GR1: *M* = 12.29, *SD* = 14.4), *U* = 312, *p* = 0.036. In parallel, the results showed no significant correlation between alternative activities carried out at home and the toddlers’ tactile exploration skills (all *ps* > 0.05).

### 3.5. Effect of Age Groups on Screen Habits and Tactile Exploration

Table 5 presented the evolution of screen habits and scores on different tactile exploration tasks, as a function of the toddlers’ age.

Screen habits. The results indicated that toddlers between 6 and 18 months old had significantly lower direct and total exposure times than toddlers between 19 and 24 months old (respectively, *t*(72) = −2.789, *p* = 0.008, and *t*(72) = −2.213, *p* = 0.031), and lower than those of toddlers between 25 and 36 months old (respectively, *t*(99) = −4.488, *p* < 0.001, and *t*(99) = −2.703, *p* = 0.008). Conversely, there were no significant differences between the groups for the background exposure time.

Three objects task. The results indicated that there were no significant differences in the total exploration time between toddlers in the two age groups. Concerning the evolution of the strategies used between age groups, the results indicated that toddlers between 19 and 24 months old used significantly fewer different strategies than toddlers between 6 and 18 months old—*U* = 372.5, *p* < 0.001—and fewer in total, *U* = 404.5, *p* = 0.002.

In addition, the results showed significant shifts in the strategies used within these two groups. Toddlers between 6 and 18 months old used the putting in mouth, throwing, and shaking strategies significantly more often than toddlers between 19 and 24 months old, while they used the pressing strategy significantly less often. The results are presented in Table 6.

Cube task. Overall, toddlers between 25 and 36 months old explored the cube significantly longer than toddlers between 19 and 24 months old—*U* = 1306, *p* = 0.037—as well as longer than toddlers between 6 and 18 months old, *U* = 1806.5, *p* < 0.001. They also explored more different faces—*U* = 1603.5, *p* = 0.007—and more faces in total—*U* = 1614, *p* = 0.005—than did the toddlers between 6 and 18 months old. Toddlers between 19 and 24 months old also tended to explore longer—*U* = 858.5, *p* = 0.053—and to explore more faces in total—*U* = 857, *p* = 0.053—than toddlers between 6 and 18 months old.

## 4. Discussion

In the present study, we investigated whether screen-use habits were related to the development of 6- to 36-month-old toddlers’ tactile exploration skills (assessed by placing various objects in front of children, which they could then observe and explore as they wished), as well as possible links with different factors such as the practice of interactive co-viewing during screen exposure, alternative activities carried out at home, and families’ socio-economic level.

First, we found that the toddlers’ screen exposure time increases with age, in line with the results in the existing literature [59], although the actual times were very low compared to screen times reported in other studies (e.g., Refs. [60,61]). The use of screens by parents of toddlers in their presence seems, however, to be stable over the years, at over an hour and a half a day. We also found that tactile exploration skills develop smoothly and habitually in children, following the developmental curves usually observed at this age (e.g., Ref. [27]). The older the children are, the longer and more thoroughly they explore the objects in front of them, thereby confirming our first hypothesis. The results of the cube task indicated that older toddlers explored the cube significantly longer than younger toddlers, and that they also explored more different faces and more faces in total. As they get older, toddlers’ attentional capacities develop, and they are more able to stay focused on the same object for longer periods [62]. In addition, very young infants are very selective in their attention, and they may have difficulty disengaging from salient stimuli. This could explain why toddlers between 6 and 18 months old also explored fewer faces in total, focusing more on those they were currently exploring [63]. Moreover, the results of the three objects task indicated that the exploration strategies used to discover the wooden rattle, the wrist cuddly toy, and the plastic keys become more complex as the child aged. Toddlers between 6 and 18 months old used the putting in mouth, throwing, and shaking strategies significantly more often than did the toddlers between 19 and 24 months old, while older toddlers used the pressing strategy significantly more often. These results are in line with those of the scientific literature on this topic, as well as those regarding the development of tactile exploration strategies in toddlerhood [27,64]. Putting objects in the mouth and shaking them are the first strategies used in the developmental process, while the pressing strategy requires more advanced fine motor skills (e.g., pressing the little button in the right place, triggering the music coming out of the plastic keys), skills which emerge later in the development process. Furthermore, the wariness phenomenon, usually noted for new objects at the age of 9 months old [23], was not observed in our results: the objects used were sufficiently familiar and similar to the toddlers’ everyday objects to conclude that these results reflect their real tactile exploration skills.

Concerning the effects of screen habits and alternative activities on tactile exploration, the results were in line with our second hypothesis: screen habits may have a role to play in the development of toddlers’ exploration skills and the relevance of the strategies used. First, toddlers between 6 and 18 months old who had not yet been directly exposed to screens explored the red face longer than toddlers who had already been exposed to screens. This face is one of the most complex to explore, requiring considerable fine motor skills. To explore it correctly, toddlers need to be able to insert their finger into a small hole and make circles with it to turn the rotating dial. The toddlers at this age who have never been exposed to screens spend more time discovering their environment, and the development of their fine motor skills enables them to explore the same faces as the older toddlers. Second, the results indicated that toddlers between 25 and 36 months old with greater direct screen exposure time explored the purple face longer than those with shorter exposure times. This face is the least explored by the entire sample, as well as the easier face. As the displacement hypothesis postulates, screen use reduces time and opportunities for toddlers to explore their environment. This may make them less accustomed to complex objects, so exploring complicated faces may be too costly for them. This hypothesis is confirmed if we look at the relevance of the strategies used by toddlers between 19 and 24 months old: the greater the background and total exposure time, the more often the shaking and throwing strategies were employed. Therefore, toddlers with more exposure to screen time more often use the same strategies as children between 6 and 18 months old, strategies which are less complex and less relevant to their age group for discovering their environment and learning about the physical properties of objects. Yet this knowledge is needed to more accurately predict the actions and behaviors of adults when confronted with these same objects, as well as for appropriate environmental interactions [65]. Finally, the results indicated that toddlers between 25 and 36 months old with greater background and total exposure time explored more faces in total. Normally, during the preschool years, attention control at a higher level becomes more important: the ability to engage in complex activities develops further and supports the maintenance of attention [62]. However, television in the background can interfere with this process, pulling the child away from the activity at hand [54]. This makes it difficult for them to stay focused on the same task for long. This can be seen in the results, which showed that these toddlers changed faces more often during exploration. When television is on more often in the background, the children are less attentive to their play, to their bodies, or to interactions with the environment [66], and all their exploration processes are hampered. However, in our sample, the most exposed toddlers between 25 and 36 months old are those with a lower socio-economic level, and these latter results are no longer significant after controlling for this factor.

These results are in line with our third hypothesis. Generally, families with a lower socio-economic level have a more positive attitude towards screens, use them more on a daily basis, and have more lax rules regarding home life and screens [67,68]. These are also families who have less income available to spend on offering their children alternative and cultural activities [69] and who use screens more often as a babysitter [70]. The differences in the development of the tactile exploration skills of these toddlers may therefore also be due to the fact that toddlers with greater screen exposure time have lower socio-economics levels and thus, are less stimulated by their parents and engage in fewer daily alternative activities. Moreover, the results also indicated that toddlers between 25 and 36 months old who did not engage in interactive co-viewing during screen exposure explored the orange face longer. Yet, this is the face most explored by toddlers between 6 and 18 months old. Moreover, toddlers between 19 and 24 months old who participate in a greater number of different alternative activities during the day used the pressing strategy, as well as the turning and observing strategy, more often. These are the most complex and age-appropriate strategies for discovering and understanding their environment. Toddlers who are more stimulated daily by their parents and who participate in more alternative activities develop better tactile exploration skills. Parental support and a wide range of daily activities are vital to child development. Toddlers need the support and guidance of their parents to grow harmoniously and develop all their skills. They need help to understand the world around them, whether this involves understanding the content on a screen or discovering everyday objects [71,72]. Screens are not solely responsible for altering the development of tactile exploration skills, but they are complementary to a lack of access to toys, leisure activities, and environmental interaction. When these different factors are combined, the effects of screens are diminished, or even reversed [58].

However, there are several limitations to this study, particularly with regard to measurement methods. First, data on screen-use habits and alternative activities were collected using a questionnaire. Parental self-reports might not be the best way to collect data on toddlers’ screen exposure because social desirability may lead parents to underestimate the total number of minutes they spend in front of screens [73,74]. Moreover, the list of alternative activities given by parents may not be exhaustive: when recalling a list of items (in this case, the daily activities performed by the toddler), individuals tend to remember more of the first and last items than those in the middle (i.e., primacy and recency effect; e.g., Ref. [75]). Parents may then forget to mention certain daily activities performed by their toddler and list only the main activities they have in mind. Additionally, all toddlers in the sample attended daycare at least once a week. Consequently, they are engaged in a significant number of alternative and manual activities, which are not accounted for here. The potential effects of screen exposure on toddlers’ exploration skills, underpinned mainly by the displacement hypothesis and the interferences due to background exposure, could thus be counterbalanced by the activities carried out daily at the daycare. Research indeed suggests that children who attend childcare services are less likely to experience developmental vulnerabilities [76,77]. Furthermore, it has been shown that toddlers develop a secure attachment with their daycare staff [78], which enables them to increase their exploratory behaviors. Finally, the assessment of tactile exploration abilities in structured play observations presents certain limitations. Although no specific instructions were given to the toddlers, they were seated at a table with specific objects to explore. They were therefore not free to explore their environment and surrounding objects as they wished, whereas toddlers have been shown to interact with a large number of different objects over short periods of time. Certain exploration characteristics cannot be assessed in such a context (e.g., which objects the child would have chosen if presented with more options). Although the objects used in our experimental tasks were sufficiently familiar to the toddlers being assessed, observing toddlers in their familiar setting, where they spend most of their waking hours, would provide more precise data on their spontaneous exploration abilities and offer more developmental insights [79]. Beyond the limitations of the measurement methods, a major limitation of this study lies in the fact that the average socio-economic level of the toddlers is the entrance level to the Swiss upper-middle class. Generally, families from the most disadvantaged classes are those who have a more positive attitude towards screens and who admit to setting fewer rules limiting screen exposure times [67]. These are also families with less economic and cultural capital to carry out a greater number of daily alternative activities. Additionally, all the parents in our sample were native French speakers, which reduces the role of diversity, whereas it has been shown that the cultural environment in which children grow up also plays a role in screen habits [80]. For future research, it would therefore be interesting to also analyze the effects of screen habits and alternative activities on tactile exploration skills in toddlers from lower socio-economic classes and to employ a sample more representative of the general population.

In all instances, on the basis of the precautionary principle, international guidelines recommend banning screens before the age of one-and-a-half to two years [56,81,82]. Several problems emerge with these recommendations: only few parents follow them [68]; they tend to address concerns more widely shared by higher socio-economic families, who are often in a better position to implement them [83]; and screens have acquired an increasingly central place in our daily lives. Society is hyperconnected, and we all use screens on a daily basis. Moreover, families from lower socio-economic backgrounds have a more positive view of screens and tend to use them more. Banning them from their daily lives would not be effective. On the other hand, offering effective support systems to help parents regulate their children’s use of screens is a successful strategy. Some interventions have already been tested to help and guide parents in the appropriate use of screens. The Intervention Nurses Start Infants Growing on Healthy Trajectories (INSIGHT), for example, was created to raise parents’ awareness of screen use, the risks of television in the child’s room and at mealtimes, and the benefits of interactive play in the parent–child relationship [84]. The results showed that parents who benefited from the intervention were more inclined to comply with the recommendations: fewer toddlers were exposed early compared with those in the control group, and the duration of the exposure was shorter. Furthermore, it might also be interesting to raise parents’ awareness of their own use of screens in the presence of their infant. Background exposure time also seems to have an effect on toddlers’ development and is an integral part of their daily lives. Finally, an interventional approach could be proposed, suggesting a wide range of alternative activities that would not be costly in terms of funding and therefore, would be accessible to everyone. Parents could replace some screen time with activities such as crafts, cooking, drawing, and outdoor play with toddlers. All these activities are easy to carry out and support the development of tactile exploration skills [85].

Beyond the implementation of such interventions, it would also seem pertinent to conduct a longitudinal study to investigate the long-term effect of early screen exposure on visuo-tactile exploration skills. It could be that a deficit in the development of these skills, primarily observed in younger toddlers, is mirrored later in regards to overall development (e.g., the cascade models; Refs. [86,87]), thus impacting the child’s general progress. Specifically, Rose et al. have shown that cross-modal tactile vision (a competency used by toddlers in our two experimental tasks) at 12 months old predicted various outcomes, including IQ scores, language competence, and perceptual speed, in children ten years later [88].

## 5. Conclusions

To develop harmoniously, toddlers need to open up to the world around them and to their immediate environment, and this occurs mainly through tactile exploration. Manual interactions with objects are therefore essential and contribute to cognitive, sensory, identity, social, and emotional development. Nevertheless, the development of certain tactile exploration skills seems to be influenced by the presence of screens in toddlers’ daily lives, but this development is also influenced by factors such as socio-economic level and alternative activities carried out daily at home. Encouraging interactive co-viewing and providing accessible, low-cost alternatives to screen time could mitigate the adverse effects observed. To be effective, interventions should therefore focus on educating parents about the developmental benefits of reducing screen time and increasing engagement in simple, low-cost alternative activities.

## Figures and Tables

**Table 1:** Screen exposure time in minutes per day for subgroups of each age group.

Measures	6–18 Months		19–24 Months		25–36 Months	
	GR0	GR1	GR0	GR1	GR0	GR1
Direct screen exposure time (min/day)	-	20 (17)	8 (9)	88 (43)	14 (10)	98 (66)
Background exposure time (min/day)	55 (14)	180 (106)	61 (17)	237 (95)	61 (23)	185 (72)
Total screen exposure time (min/day)	58 (13)	173 (115)	75 (30)	290 (126)	77 (26)	252 (82)

**Table 2:** Three objects task and cube task scores, depending on screen habits for each subgroup (GR0 vs. GR1) of 6- to 18-month-old toddlers.

Measures	Direct Screen Exposure Time		Background Exposure Time		Total Screen Exposure Time	
	GR0 (N = 27)	GR1 (N = 13)	GR0 (N = 31)	GR1 (N = 9)	GR0 (N = 29)	GR1 (N = 11)
Three objects task						
Total exploration time	72.93 (35.366)	56.93 (43.005)	63.81 (34.527)	78.80 (48.830)	64.83 (34.817)	73.83 (46.993)
Number of different strategies used	3.70 (0.912)	3.64 (1.336)	3.77 (1.055)	3.40 (1.075)	3.72 (0.996)	3.58 (1.240)
Total number of strategies used	12.44 (5.308)	9.57 (4.957)	11.23 (4.710)	12.20 (7.115)	11.14 (4.711)	12.25 (6.717)
Cube task						
Total exploration time	34.93 (28.524)	35.92 (28.652)	36.77 (28.941)	30.00 (26.377)	36.66 (28.498)	31.55 (28.398)
Number of different faces explored	2.67 (1.519)	2.38 (1.121)	2.55 (1.410)	2.67 (1.414)	2.62 (1.425)	2.45 (1.368)
Total number of faces explored	4.00 (2.512)	3.77 (1.922)	4.16 (2.437)	3.11 (1.691)	4.21 (2.426)	3.18 (1.888)

**Table 3:** Three objects task and cube task scores, depending on screen habits for each subgroup (GR0 vs. GR1) of 19- to 24-month-old toddlers.

Measures	Direct Screen Exposure Time		Background Exposure Time		Total Screen Exposure Time		Interactive Co-Viewing	
	GR0 (N = 26)	GR1 (N = 8)	GR0 (N = 23)	GR1 (N = 11)	GR0 (N = 23)	GR1 (N = 11)	GR0 (N = 23)	GR1 (N = 11)
Three objects task								
Total exploration time	73.24 (45.364)	85.67 (65.662)	75.87 (51.036)	77.91 (52.506)	73.27 (48.133)	82.50 (56.880)	78.95 (51.980)	72.08 (50.267)
Number of different strategies used	2.80 (1.000)	2.78 (1.202)	2.65 (0.775)	3.09 (1.466)	2.64 (0.790)	3.08 (1.379)	2.82 (1.006)	2.75 (1.138)
Total number of strategies used	7.76 (4.352)	7.22 (3.456)	7.22 (3.977)	8.45 (4.390)	7.00 (3.928)	8.75 (4.309)	7.36 (3.606)	8.08 (4.999)
Cube task								
Total exploration time	43.35 (28.700)	61.25 (17.556)	47.26 (28.881)	48.18 (25.206)	45.26 (28.403)	52.36 (25.664)	50.70 (28.938)	41.00 (23.639)
Number of different faces explored	2.88 (1.608)	3.50 (1.690)	3.00 (1.624)	3.09 (1.700)	3.00 (1.567)	3.09 (1.814)	3.09 (1.649)	2.91 (1.640)
Total number of faces explored	4.92 (3.084)	6.38 (2.446)	5.17 (3.157)	5.45 (2.697)	5.13 (3.079)	5.55 (2.876)	5.52 (3.146)	4.73 (2.649)

**Table 4:** Cube task scores, depending on screen habits for each subgroup (GR0 vs. GR1) of 25- to 36-month-old toddlers.

Measures	Direct Screen Exposure Time		Background Exposure Time		Total Screen Exposure Time		Interactive Co-Viewing	
	GR0 (N = 44)	GR1 (N = 17)	GR0 (N = 42)	GR1 (N = 19)	GR0 (N = 40)	GR1 (N = 21)	GR0 (N = 26)	GR1 (N = 35)
Cube task								
Total exploration time	57.76 (29.487)	68.44 (26.45)	57.96 (31.509)	66.87 (21.397)	59.31 (31.536)	63.45 (23.410)	63.96 (27.281)	58.34 (30.150)
Number of different faces explored	3.20 (1.440)	3.88 (1.111)	3.17 (1.447)	3.89 (1.100)	3.15 (1.477)	3.86 (1.062)	3.38 (1.499)	3.40 (1.311)
Total number of faces explored	5.32 (3.079)	6.24 (2.306)	5.05 (4.154)	6.74 (1.790)	5.03 (3.190)	6.62 (1.884)	5.50 (2.929)	5.63 (2.911)

**Table 5:** Evolution of screen habits and tactile task scores as a function of age groups.

Measures	6–18 Months	19–24 Months	25–36 Months	*p*
Screen habits				
Direct screen exposure time (min/day)	6 (13)	27 (40)	38 (52)	0.001
Background exposure time (min/day)	83 (72)	118 (100)	100 (72)	0.177
Total screen exposure time (min/day)	90 (79)	145 (126)	137 (98)	0.031
*Three objects task*				
Total exploration time	67.46 (38.38)	76.53 (50.723)	-	0.602
Number of different strategies used	3.58 (1.059)	11.46 (5.311)	-	<0.001
Total number of strategies used	11.46 (5.311)	7.62 (4.09)	-	0.002
*Cube task*				
Total exploration time	32.25 (28.2)	47.56 (27.364)	60.74 (28.863)	<0.001
Number of different faces explored	2.57 (1.394)	3.03 (1.623)	3.39 (1.382)	0.029
Total number of faces explored	3.92 (2.314)	5.26 (2.978)	5.57 (2.895)	0.018

**Table 6:** Evolution of the strategies used between age groups.

	6–18 Months	19–24 Months	U	*p*-Value
Putting in mouth	2.12 (2.704)	0.15 (0.500)	350.5	<0.001 **
Caressing	0.63 (0.968)	1.24 (1.689)	834.5	0.107
Throwing	2.37 (3.144)	0.79 (2.071)	422.5	<0.001 **
Pressing	1 (1.449)	1.91 (1.848)	902	0.022 *
Turning and observing	2.8 (1.965)	2.62 (2.015)	654.5	0.646
Shaking	2.54 (2.430)	0.91 (1.505)	385.5	<0.001 **

*Note.* * indicates *p*-value < 0.05; ** indicates *p*-value < 0.01.

## Data Availability

The data presented in this study are available on request from the corresponding author. The data are not publicly available due to privacy restrictions.
